# “I’m outta here!”: a qualitative investigation into why Aboriginal and non-Aboriginal people self-discharge from hospital

**DOI:** 10.1186/s12913-021-06880-9

**Published:** 2021-09-03

**Authors:** Deborah A. Askew, Wendy Foley, Corey Kirk, Daniel Williamson

**Affiliations:** 1 Primary Care Clinical Unit, The University of Queensland, Royal Brisbane & Women’s Hospital, Level 8, Health Sciences Building, Qld 4029 Brisbane, Australia; 2 Southern Queensland Centre of Excellence in Aboriginal and Torres Strait Islander Primary Health Care, Metro South Hospital and Health Service, 37 Wirraway Parade, 4077 Inala, Qld Australia; 3 Aboriginal and Torres Strait Islander Health Division, Queensland Health, 33 Charlotte Street, Qld 4001 Brisbane, Australia

**Keywords:** Self-discharge, Discharge against medical advice, Culturally safe health care, Person centred care, Patient rights

## Abstract

**Background:**

Occasions of self-discharge from health services before being seen by a health profession or against medical advice are often used by health systems as an indicator of quality care. People self-discharge because of factors such as dissatisfaction with care, poor communication, long waiting times, and feeling better in addition to external factors such as family and employment responsibilities. These factors, plus a lack of cultural safety, and interpersonal and institutional racism contribute to the disproportionately higher rates of Indigenous people self-discharging from hospital. This qualitative study aimed to increase understanding about the causative and contextual factors that culminate in people self-discharging and identify opportunities to improve the hospital experience for all.

**Methods:**

Semi-structured interviews with five Aboriginal and/or Torres Strait Islander (hereafter, respectfully, Indigenous) people and six non-Indigenous people who had self-discharged from a major tertiary hospital in Brisbane, Australia, were audio-recorded, transcribed and thematically analysed.

**Results:**

Study participants all respected hospitals’ vital role of caring for the sick, but the cumulative impact of unmet needs created a tipping point whereby they concluded that remaining in hospital would compromise their health and wellbeing. Five key categories of unmet needs were identified – the need for information; confidence in the quality of care; respectful treatment; basic comforts; and peace of mind. Although Indigenous and non-Indigenous participants had similar unmet needs, for the former, the deleterious impact of unmet needs was compounded by racist and discriminatory behaviours they experienced while in hospital.

**Conclusions:**

Respectful, empathetic, person-centred care is likely to result in patients’ needs being met, improve the hospital experience and reduce the risk of people self-discharging. For Indigenous people, the ongoing legacy of white colonisation is embodied in everyday lived experiences of interpersonal and institutional racism. Racist and discriminatory behaviours experienced whilst hospitalised are thus rendered both more visible and more traumatic, and exacerbate the deleterious effect of unmet needs. Decreasing self-discharge events requires a shift of thinking away from perceiving this as the behaviour of a deviant individual, but rather as a quality improvement opportunity to ensure that all patients are cared for in a respectful and person-centred manner.

**Supplementary Information:**

The online version contains supplementary material available at 10.1186/s12913-021-06880-9.

## Background

Although relatively uncommon, occasions of people self-discharging from health services before being seen by a health profession or against medical advice are associated with increased patient morbidity and mortality, and greater risk of hospital readmission with associated personal, health system, and societal costs [1]. People self-discharge because of dissatisfaction with care, a lack of communication, long waiting times, feeling better, and family and work responsibilities [2–5]. Unsurprisingly, it is therefore defined as a healthcare quality problem [1]. It is an indirect indicator of how well hospital services meet their in-patients’ needs and is a proxy measure of cultural safety for ethnic minority groups and Indigenous peoples [6].

People who self-discharge are generally perceived as poor decision makers and castigated, either to their face or in their medical records, for behaving inappropriately [7]. The very language used is adversarial and denotes a hierarchy where health professionals’ decisions and opinions take precedence over those of their patients, rendering patients relatively powerless. If a patient does decide to self-discharge, their reasoning is questioned, and they are typically derided as irrational and labelled as demanding [7]. However, competent patients in Australia and elsewhere have the legal right to make decisions about their healthcare, including the right to self-discharge from a healthcare facility [8]. Clearly, the pejorative and paternalistic portrayals of people who self-discharge conflict with these rights.

In Australia’s major cities, Aboriginal and Torres Strait Islander (hereafter, respectfully, Indigenous) people are hospitalised at approximately the same rate as their non-Indigenous counterparts, but in 2017 were 5.4 times more likely to self-discharge [9]. Indigenous people describe an absence of cultural safety in hospitals, experiences of isolation and loneliness, mistrust in the health system, and, for some, association of hospital with death as key reasons why they self-discharge [3, 6, 10–12]. Institutional racism, defined as race-based differential access to society’s goods, services, and opportunities, contributes to the lack of cultural safety and the disproportionate representation of Indigenous people in Australia’s self-discharge statistics [13, 14]. To understand why this is so, previous research has created epidemiological profiles to identify which sub-groups of Indigenous patients are most likely to self-discharge, or focussed qualitative investigations on factors associated with self-discharge in selected population subgroups. In contrast, this research aimed to increase understanding about the causative and contextual factors that culminate in Indigenous and non-Indigenous people self-discharging and identify opportunities to improve the hospital experience for all.

## Methods

### Study design

This qualitative phenomenological study aimed to describe the lived experiences of people who had self-discharged from the Princess Alexandra Hospital (PAH), Brisbane, Australia. The PAH is a major tertiary health care centre and provides care in all major adult specialities except obstetrics [15]. Phenomenological research aims to elucidate the meaning, structure and essence of a phenomenon, from the perspectives of people who have personally experienced it [16]. This approach assumes a commonality in experiences, and the analysis aimed to identify and explore that commonality [16]. For this research, the experiences shared by participants are both the self-discharge event and the experience of being an inpatient at the PAH.

Researchers from the Southern Queensland Centre of Excellence in Aboriginal and Torres Strait Islander Primary Health Care (Inala COE) led this research (DA, WF, CK), in partnership with the Cultural Capability team from the then Aboriginal and Torres Strait Islander Health Branch (DW), Queensland Health. DA and WF, both female and both with PhDs, were qualitative researchers with experience in researching Aboriginal and Torres Strait Islander health. CK, a male, was a novice researcher, and DW, male, was an epidemiologist with broad health system knowledge and understanding. CK is an Aboriginal man, and DA, WF & DW are all non-Indigenous. All researchers had, at some stage of their lives, been admitted to a hospital, thus they and the research participants had a shared experience of being a hospital patient.

### Study sample selection and participant recruitment

Study participants were 18 years and older and were recorded in the PAH discharge database as having self-discharged from the PAH between 1 July 2013 and 28 February 2014. The discharge database records occasions of self-discharge as a standard category of discharge disposition. Patients with a principal diagnosis involving substance abuse or serious mental illness were not eligible due to the associated complexity of factors that increase their risk of self-discharge [17–19].

To create the list of potential participants, Indigenous people who had self-discharged were initially identified – Indigeneity is also recorded in the discharge database. Non-Indigenous people who had self-discharged were then matched to the Indigenous people by discharge division, principal diagnosis, age, and sex in an attempt to maximise the structural similarities of participants’ inpatient experiences.

Using patient contact details held by the PAH, CK, the Aboriginal Research Officer (ARO) working on this study, commenced telephoning the people on the list. He used a variety of telephones, including the office landline, a work mobile telephone and his personal mobile telephone due to awareness that many people are reluctant to answer unknown or private telephone numbers. When he connected with the potential participant, he explained the study’s rationale and approach, how their contact details had been obtained, and reassured them that the research and researchers were independent to the hospital. These telephone calls were the first contact between the researchers and the participant, except for one participant who was from the same Aboriginal and Torres Strait Islander community as CK and known to each other. Interviews were conducted at a time and place of maximum convenience for the participant. Participation was explained again, participants were invited to ask questions about the research, and informed written consent was obtained prior to any data collection. Participants received an AUD$25 supermarket voucher as acknowledgment of their contribution to the research.

The PAH’s two Indigenous Hospital Liaison Officers (HLOs) were also interviewed as their roles aim to support Indigenous people while they are hospitalised, and to decrease incidents of Indigenous people self-discharging. The interview was guided by an interview schedule developed for this study and provided as Additional File 1.

### Data collection

Where possible, interviews were conducted face-to-face by the ARO (CK) and a second researcher (DA or WF) in participants’ homes or other location that best suited the participant. Telephone interviews were conducted if face-to-face interviews were not possible. The interviews were guided by an interview schedule that contained the broad topics to be covered. The schedule was developed for this study following a review of the extant literature, and was pilot tested within the research team. No alterations to the broad topics were required. The patient interview schedule is provided as Additional File 2. Interviews were, with permission, audio-recorded and transcribed verbatim by an external company. Transcripts were checked against the audio-recordings, corrected and de-identified prior to analysis. Transcripts were not returned to participants for checking, and repeat interviews did not take place.

Participants’ hospital medical records were reviewed to compare the documented version of the self-discharge event with the participant’s account.

### Data analysis

CK, WF and DA debriefed after each interview by sharing their reflections of the participant’s hospital experience and factors contributing to the decision to self-discharge, and compared these reflections with prior interviews. Additionally, the actual processes of the interviews were discussed to facilitate incorporation of any lessons learnt into subsequent interviews. Notes taken during these post-interview debriefs were included in the analysis.

Interview transcripts were read and re-read by CK and WF to identify themes and sub-themes using thematic analysis [20]. Following identification of themes, the transcripts were re-read by CK, WF and DA to ensure that the richness of participants’ narratives had not been diminished by researcher-imposed fragmentation of their accounts into themes. Themes were further refined at this stage. Because the researchers had all been hospitalised, issues discussed by the participants were not unfamiliar and the researchers could empathise with the frustrations expressed by participants. Ensuring that all researchers were involved in developing and refining the themes ensured that the previous inpatient experiences of any one of the researchers did not overly influence the analysis. CK’s involvement in the analysis ensured that Indigenous participants’ accounts were interpreted through an Indigenous lens and the Indigenous voices were privileged in the analysis.

## Results

### Participants

During the nine-month study period, a total of 484 people were recorded as having self-discharged from the PAH, of whom 21 had subsequently passed away. Of the remaining 463, 61 (13.2 %) identified as Indigenous people. Following matching to their non-Indigenous counterparts, a list of 90 potentially eligible study participants was derived. On reviewing the principle diagnoses, six people were excluded (illicit substances (*n* = 1), alcohol abuse (*n* = 4), and suicidal ideation (*n* = 1)), resulting in a list of 84 people who were eligible to participate.

**Fig. 1:**
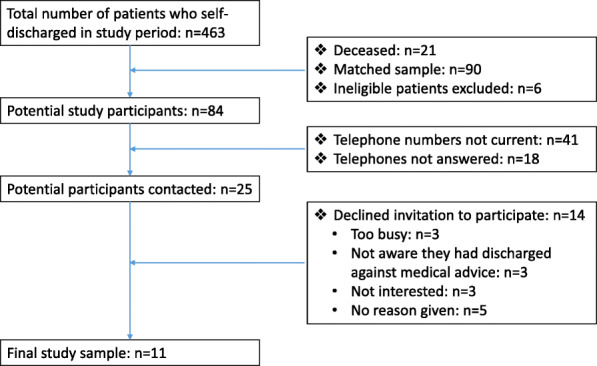
Recruitment flow chart

After more than 200 attempts to contact the people on the list by telephone, CK had spoken to 25 (30 %); 11 of whom agreed to participate –an effective recruitment rate of 44 % (11/25) (Fig. 1). All but one interview was conducted face-to-face at the Inala COE or in the participants’ home, with the exception being conducted over the telephone at the request of the participant. Participants could have another person present at the interview if they wished. None did so. Interviews lasted between 20 and 40 min; the telephone interview was among the shorter of the interviews.

Table 1 presents selected characteristics of participants and their hospitalisation. Their mean age was 49.5 years (range, 40–63 years). The 3 participants who had been admitted more than 100 times were all receiving thrice-weekly haemodialysis: each dialysis episode is recorded as an admission. All Indigenous participants and one non-Indigenous participant had previously self-discharged.

**Table 1:** Selected characteristics of study participants

Participant ID^a^	Sex	Age (years)	Length of Stay prior to self-discharge (days)	Number admissions to the PAH since 1992^b^	Discharge Unit
IDI1	Female	63	1	3^c^	General medical
IDI2	Male	43	1	26^c^	Cardiology
IDI3	Female	56	5	198^c^	Renal
IDI4	Female	59	4	985^c^	Cardiology
IDI5	Male	56	1	8^c^	Gastroenterology
NDI1	Male	43	1	9	General medical
NDI2	Female	40	1	276	Endocrinology
NDI3	Male	45	1	45^c^	Renal
NDI4	Female	42	1	1	Cardiology
NDI5	Male	45	1	1	Colorectal
NDI6	Male	52	1	1	Orthopaedic

^a^ID code: *IDI *Indigenous participant, *NDI *non-Indigenous participant

^b^Digitalised patient records at the PAH date back to 1992

^c^participant reported previous instances of self-discharge

### Why people self-discharged

The cumulative impact of unmet needs was at the core of each participant’s account of why they self-discharged. These needs have been grouped into five broad, but interrelated themes: the need to know and the need to be heard; the need for confidence in the quality of care; the need to be treated with dignity and respect; the need for nourishment, sleep and stimulation; and the need for peace of mind.

#### The need to know and the need to be heard

All participants reported a lack of effective communication. Frequently they did not understand what health professionals said, particularly when specialised medical terminology was used. They felt that the health professionals had not spent sufficient time explaining their diagnoses or planned investigations or procedures so they actually understood what was planned to occur during their hospitalisation. Participants recounted frequently asking for more information or clarification, and also feeling that health professionals deliberately withheld information about their diagnoses, the likely duration of their hospitalisation, why various investigations were conducted, and the timing of procedures during their admission. Thus, the relative powerlessness of patients compared to hospital staff was reinforced through this perceived withholding of information. One participant recounted the frustration of having a procedure rescheduled three days in a row to be finally told that the necessary equipment to do the procedure was not available….

*“…I waited patiently but then, no, nothing happened … so then they said, ‘rightio, tomorrow morning first thing’ … tomorrow morning came, nothing happened, no doctors, no nothing. I asked and asked and the nurses said, “Oh, yeah, we’ll send the doctor around” … and the nurses come and they [said], “yes, oh, the doctors were in a hurry, they’ve got other patients … they’ll put you in tomorrow” … this went on for three days and they put me in this little ward where you all wait to go into the area … it was Friday … I was watching the clock and 3 o’clock in the afternoon and I said, “Youse aren’t doing it, are you?“ “Oh, we haven’t got the equipment.“ I said, “You know what? Youse are a bunch of arseholes,“ I said, “because youse could have told me that in the first place.“ “Oh, but you don’t have to get upset.“ they said. I said, “Of course I’m bloody upset.“ I was swearing and everything. I said, “I’m outta here … so I just packed my bag and walked out…” (IDI4)*.

The communication barrier between health professionals and participants was also apparent in how participants responded to notification of their imminent discharge. For some participants, being told they were to be discharged was interpreted as being told they were free to go. In reality, there was often a lengthy delay between the telling and the completion of paperwork and receipt of medication which had to occur prior to the official discharge. Thus, some people were recorded as self-discharging whereas they believed they had been discharged. Other study participants recounted being informed they would be discharged the following day, but uncompleted paperwork, lack of communication between staff, or additional investigations precluded this from occurring. For study participants, the mixed messages and the lack of adequate communication coupled with apparent withholding of information contributed to an array of emotions such as anger, frustration, a sense of being targeted, and feeling demoralised and disheartened. These emotions, and the preceding events, triggered their decisions to self-discharge.

#### The need to be confident about the quality of care

Perceptions of a lack of communication between hospital staff and perceived disorganisation at the hospital level led some participants to question the quality of care they received during their admission. Some participants recounted occasions they had received conflicting information from different staff members. Two participants believed they had been wrongly diagnosed, and others recounted how their knowledge about their own bodies was disregarded and ignored, as if their knowledge was not as valid or as important as that of the health professionals. Similar to the participant quoted above, others also had planned procedures cancelled at the last minute because test results were not available. This sense of disorganisation at the personal and system level eroded participants’ confidence in the quality of care they received and gave rise to concerns about potential deteriorations in their health.

As noted above, most study participants had been admitted to the PAH more than once, with some having had numerous admissions over the years. These participants reflected on both material and attitudinal changes that had occurred over the years, such as the bedside television no longer being free, patients no longer getting taxi vouchers, and not feeling supported and cared for while in hospital, with one stating that ‘*no one seems to care anymore*’(IDI4).

Compounding these doubts about the quality of care for the Indigenous participants was their perception of being subjected to discriminatory treatment, with one stating “…*it’s just the things on the side [that you notice]…”* (IDI5). One Indigenous participant believed she had been subjected to inequitable access to transportation compared to non-Indigenous patients. Another recounted how she had presented to the hospital with chest pains, and a doctor observed her scratching her legs and diagnosed her with scabies. This participant spoke of being isolated without explanation until a food-service staff member informed her of the scabies diagnosis. The participant felt the doctor held beliefs about Aboriginal people based on negative stereotypical portrayals, which caused the doctor to automatically associate her Aboriginality and her scratching with a diagnosis of scabies. For this participant, the diagnosis and the isolation was reminiscent of her experiences of living through the eras of government policies of segregation and assimilation….

*You get them vibes … [felt] like we were back in the 60 or 70 s … it all came back again* … *because I’m Black, too, most likely, you know, scabies there … you get that all the time…* (*IDI1*).

The HLOs also recounted episodes where they saw Indigenous patients being subjected to discriminatory and racist behaviours, including other patients staring and whispering, or staff being judgemental and intolerant. The HLOs believed that self-discharges could be prevented by hospital staff providing person-centred care and being attuned to individual needs of each patient. They spoke of the negative consequences for patients’ wellbeing of overhearing staff denigrating them to other staff, or staff not caring about patient’s needs… *“just [staff] being a little bit aggressive or grumpy or I haven’t got time for you today; what do you want? You’re ringing that buzzer again? What are you doing? So, we get a lot of that…”(HLOs).* The HLOs did acknowledge that hospital staff are busy, and have stressful and challenging jobs but that the provision of attuned and compassionate care is required, encapsulated in the following extract….

*“…I know they’re busy with most of the other patients, but you have to be present with every contact, every attendance that you’re in, and you have to listen to what that person’s request is. So you’ve got to get rid of any conversation in your head about what your perception of this person is and just go and listen. So you can’t go in with preconceived … ideas… but the simplest thing is listening … clear your head … stop, get rid of the internal chatter, go in and BE with this person…” (HLOs)*.

#### The need to be treated with dignity and respect

A recurrent theme was the perception that hospital staff lacked respect, humanity, compassion and basic kindness in their treatment and attitudes towards patients. A number of participants recalled episodes of staff being rude and disparaging, talking to them in a patronizing or condescending manner and ignoring them when they requested assistance or information. Participants described occasions where their relative powerlessness had been reinforced by the hospital staff, with one recounting how one doctor had given her a pass-out to spend Christmas Day with her family, and then another doctor ordered tests to be done on Christmas Day, therefore preventing her from going home. Her frustration and anger is evident in this extract….

*“…I still don’t know really why I had to have those tests that [Christmas Day] … she wanted to prove her authority, I could see that in the end. She was determined to make me stay there. And I was just as determined … to go…” (IDI3)*.

Another participant, who was not from Brisbane, recounted how staff appeared to arbitrarily interpret and apply hospital policies. On the first night of her admission, the ward staff allowed her husband to stay at her bedside throughout the night. However, without explanation, he was not permitted to stay at her bedside on the second night. Indeed, he was threatened with forcible removal by security if he did not go. The participant recalled begging ward staff to allow her husband to stay, but they would not allow it. Additional factors contributing to the stress of the situation was that the husband could not go home, had no money to pay for accommodation, and no friends with whom he could stay. The participant and her husband could not understand why the hospital policy was interpreted and enforced differently by different staff members, and felt the nursing staff capriciously determined how they would interpret policies and procedures. She stated….

*“… [they] didn’t show any … no compassion at all and really, I was quite shocked, okay … no compassion, no consideration of people’s personal circumstances…” (NDI4)*.

An essential element of treating people with respect and dignity is respecting people’s identity. For Indigenous people, their Indigeneity is a fundamental component of who they are and how they live in the world. However, the Indigenous participants felt hospital staff perceived their Indigeneity as problematic, and a predictor of poor health related behaviours and attitudes. They felt ‘labelled’ and judged by hospital staff. One Indigenous participant, a smoker, had attended the emergency department. While waiting to be assessed, he went outside for a cigarette. On his return, he found he had been labelled as self-discharged and told that “*if you’re well enough to go out for a smoke, you’re too well to be here*” (IDI2). His nicotine addiction was not acknowledged, nor were alternative sources of nicotine offered. Another Indigenous participant said that he was labelled as an alcoholic and treated with disdain, and another felt her Indigeneity negatively influenced her diagnosis and treatment.

All Indigenous participants, including the HLOs, had experienced interpersonal racism and were impacted by the institutional racism in the hospital – as one HLO stated, ‘*this is a white institution with white doctors’*. Indigenous participants felt that staff lacked knowledge and respect of Indigenous culture, with one suggesting that instances of self-discharge could be prevented with “*just a little bit more understanding cultural wise…”*(IDI3). Interestingly, the HLOs reflected that the hospital is really just a microcosm of society and therefore mirrored how racism manifests in broader society….

*I think straightaway [the Indigenous patients are] the “Other”, just like they are out in the general public. So it’s just a microcosm type effect – it’s just the hospital here, but these guys, if they haven’t had a lot to do with Aboriginal people … they only know what’s on the media … they’re going to have this … belief that this is the story and decide how to fit an Aboriginal and Torres Strait Islander person into the created story … they’ll have a look and they’ll make their judgement on how that person is going to be treated … not all, but … I think straight up, yeah, they would treat them differently from non-Indigenous. (HLOs)*

#### The need for nourishment, sleep and stimulation

Unmet basic physiological needs triggered self-discharge for some participants. Two participants described being unbearably uncomfortable in their hospital beds and others described how their financial insecurity limited their options while in hospital. For example, one participant could not afford the television and so just lay in his bed with nothing to do, and left when he felt better. Another had very particular dietary habits which meant he could not eat most of the food provided by the hospital. A staff member suggested he could purchase food from the cafeteria, however, he had insufficient money to do this. He reported being ‘famished’. He wanted to go home to eat and return the following day for the required care. This was not possible, and therefore he self-discharged.

#### The need for peace of mind

The hospital admission was but a moment in participants’ lives. For some, concerns for family left at home contributed to their self-discharge decision. One participant recounted how the image of his daughter as he was driven away in an ambulance kept replaying in his mind. He became increasingly worried about her, and discharged himself when he felt better.

*“…even when I was in there my concern was what was going on here at home. I’ve got a 15 year old daughter, mate. She was devastated when I got taken away in an ambulance…”(NDI6)*.

Another participant, a single parent, recounted having to either leave her daughter at home alone or find other family members to care for her. Concerns for her daughter’s safety had driven her to self-discharge on previous occasions….

*“…but you still worry … a couple of times I’ve had to come home because we’ve had troubles around our house…” (IDI3)*.

#### Participants’ attitudes towards hospital and self-discharging

These results need to be considered against the backdrop of participants’ fundamental belief in the importance of the hospital. They acknowledged that being hospitalised was not particularly pleasurable, with “*sick people all around*” (NDI1) and a place where “*you’re out of your comfort zone*” (NDI1). Nevertheless, they respected the hospital’s role of providing specialised health care, and all but one stated they would not have self-discharged if they still felt unwell or were in pain. Interestingly, all participants, even those who had previously self-discharged, acknowledged that patients self-discharging can be problematic.

*“…It is a problem for people to be discharging themselves from hospital. It’s not something that I would recommend to anybody. You’re in there for a reason and you need to be there. Discharging yourself against doctors’ advice is not the best option for anybody…”* NDI1.

The HLOs also viewed self-discharge as problematic, and did their best to support Indigenous patients to remain in hospital until they were discharged. The HLOs considered that broadly, Indigenous people who self-discharged fell into two groups. The first were those who had been told they were due for discharge, and simply left before all the discharge procedures have been completed. The second were those who were admitted due to acute exacerbations or complications associated with chronic diseases, and then self-discharged once the acute symptoms had resolved and they felt better. The HLOs commented that frequently people in this latter group have social circumstances that preclude adherence to a care plan, resulting in further exacerbations and admissions – creating a “*revolving door*” of admissions and self-discharge. The HLOs had observed the declining health status at each admission for people in this category, as this extract highlights….

*“… so [patient] gets treated, gets well enough, goes, but doesn’t take any medication or look after himself … so then he comes back again; he gets treated, self-discharges and it’s a complete revolving door, and each time he comes [his health] level drops …“ HLOs*.

## Discussion

Contrary to common narratives that people who self-discharge do not care about their health, study participants cared deeply about their health and wellbeing. They also respected the important role of hospital in the provision of quality health care. Participants had a holistic understanding of health and of quality health care. They expected to be treated with dignity, respect and humanity; to understand their proposed care plan while hospitalised (including any alterations or delays to that plan); and to be confident in the quality of care and the competence of the health care providers. Additionally, although being hospitalised requires people to adopt a ‘sick role’ and largely rest immobile in bed [21], not all participants were able to delegate responsibilities inherent to their social roles (for example, parenting) to others. Thus, for some, as the perceived necessity of remaining in hospital decreased (they started to feel better), these unmet responsibilities became increasingly stressful, thereby precipitating a leave event. For the Indigenous participants, their everyday lived experiences of discrimination, racism and disadvantage sensitised them to personal and institutional racism experienced whilst hospitalised, further widening the gap between the need to be treated with dignity and respect and the reality of their inpatient experience. For all participants, the cumulative effect of unmet needs created a tipping point where they believed remaining in hospital compromised their health and wellbeing. Understanding and respecting this decision requires recognition of each patient’s personhood, rather than viewing patients as powerless recipients of health care who are temporarily occupying a hospital bed [21].

For each participant, the decision to self-discharge was a rational act of reclaiming their personhood and personal power, and having agency over healthcare decisions that affect them. The power differential between participants while hospitalised and health service staff was reinforced in a multitude of ways, including the lack of timely or adequate communication, the stripping of personhood, and the assumptions made by staff that patients can simply purchase solutions to problems that arose while hospitalised. Consequently, each participant suffered as a result of their healthcare experience, whether it be through mistreatment or not being listened to, having to struggle for their health care needs and autonomy, feelings of powerlessness, or feelings of being fragmented or objectified [22]. This suffering is in stark contrast to patients’ rights to quality health care as stipulated by the Australian Commission on Safety and Quality in Healthcare [23]. Although hospitals require people to assume a patient role, with its associated characteristics of neutrality, compliance and patience, hospitalised people are still individuals. When admitted to hospital, they bring an array of life experiences, values, hopes, fears and expectations, and the decision to self-discharge needs to be considered in the context of the interaction between the individual and their environment [24]. As identified by the HLOs, the hospital staff need to be attuned to each individual patient, and be fully present with the individual during each interaction. Quality care demands this, but the reality in this study was quite different and participants were rendered powerless and suffered as a consequence of care. Therefore, the decision to self-discharge can be seen as active resistance against the disempowering and dehumanising that typically occurs within a hospital [25–27] and a reclamation of personal power.

For Indigenous Australians, the suffering caused by care while in a health care facility is exacerbated because of the divergence between Indigenous peoples’ holistic and collective understandings of health and the hegemonic western, biomedical, reductionist, and positivist view of health that informs health policy and praxis, and is embodied within health care facilities [28]. These same health care facilities, governed by western health policy, promulgate views of health that focus responsibility for health status on the individual, with disease causation largely attributed to behaviours and personal choices [28]. Thus, for health professionals trained in western medicine, the well-documented disparity in health status between Indigenous and non-Indigenous Australians can be blamed on Indigenous people’s life choices, rather than social, political, cultural and economic inequities endured by Indigenous people [28]. Thus, Indigenous people continue to suffer racism and discriminatory attitudes while hospitalised [6], are perceived as being at fault for their ill health, and are viewed as ‘non-ideal’ patients and not worthy of the same level of care as non-Indigenous patients [29]. Indigenous participants in this research spoke of isolation, loneliness, alienation, discrimination, institutional racism, and a lack of Indigenous staff who could understand and relate to them. Not surprisingly therefore, many Indigenous people have a depth of fear and anxiety that can be difficult for non-Indigenous people to understand [30].

### Strengths and limitations

Inclusion of Indigenous and non-Indigenous people who had self-discharged enabled a deeper understanding of how the decision to self-discharge is impacted by issues arising during the current hospital admission and also to events in personal, familial and community histories, and for Indigenous people, their cultural history. However, despite matching participants on discharge division, age and gender, the Indigenous participants had all previously self-discharged compared to only one non-Indigenous participant who had done so. Prior hospitalisation and prior experiences of self-discharge have been associated with self-discharge [2, 11, 31–33], and certainly some participants appeared to discuss more than one hospital admission and more than one decision to self-discharge. However, these prior experiences do not diminish the universality of participants’ unmet needs precipitating their decision to self-discharge.

Our findings are specific to one acute care hospital in Brisbane, Australia. The insights illuminated by our research may be relevant in other settings where similar contextual forces have shaped hospital care, but cannot fully account for why all patients self-discharge. A further limitation is that our participants had all self-discharged, and therefore their experiences may be negatively biased and not representative of all patients. The only hospital staff that we interviewed were the two HLOs who largely reinforced the perspectives of the Indigenous participants that institutional racism negatively impacted on Indigenous peoples’ hospital experience. Inclusion of other hospital staff in the research may have provided further insights into the contextual factors that determine how specific aspects of care are delivered, but given the focus on this research on understanding the patient experience, staff perspectives were not explored.

The use of qualitative methods to understand patients’ experiences of self-discharge was an important strength of this research. Qualitative methods have increasingly been used for public health research [24] and are particularly relevant when research questions seek meaning and understanding of social phenomena from the perspective of particular players. Use of semi-structured interviews for data collection enabled participants to tell their own stories and share their own insights. Gaining an in-depth and nuanced understanding of why people self-discharge that goes beyond the oft quoted socio-demographic risk factors provides insight into how the contextual factors cause harm to patients, and also where changes in the system could potentially improve the hospital experience and decrease self-discharge risk. Younger males, purportedly a population sub-group at high risk of self-discharge [2, 34], were under-represented in this research and therefore may be exempt from the conclusions reached. A further limitation was our reliance on contact details held by the hospital, with nearly half of the telephone numbers being disconnected. The changing communications landscape, with decreased prevalence of landlines and frequent updating of mobile phones is making phone-based recruitment increasingly difficult, and could explain the underrepresentation of younger males [35]. Interestingly, once phone contact was made with potential participants, we achieved a 44 % recruitment rate which suggests the salience of this research topic to those who had self-discharged. Although we had anticipated interviewing more people, the difficulties making contact with potential participants precluded this from occurring. Nonetheless, data saturation was achieved and further interviews were not pursued.

## Conclusions

Although self-discharge can be associated with adverse health outcomes [7], competent patients have an inalienable right to make this decision. The decision to self-discharge removes the shackles associated with the ‘patient’ label and enables recovery of a person’s individual identity. For Indigenous Australians, identity and culture are inextricably entwined. Quality healthcare for Indigenous Australians must therefore be responsive to their cultural needs. Respectful, person-centred healthcare where safety is ensured is likely to result in all patients’ needs being met and reduce occasions of patients self-discharging.

## Supplementary Information

**Additional file 1. **DAMA Study: Indigenous Hospital Liaison Office Interview Schedule

**Additional file 2.** DAMA study: participant interview schedule

## Data Availability

The data the support the findings of this study are not publically available, but are available from the COE, via the corresponding author, providing appropriate ethical and community approvals are obtained.

## Abbreviations

ARO: Aboriginal Research Officer
CK: Corey Kirk
DA: Deborah Askew
DW: Daniel Williamson
HLO: Hospital Liaison Officer
IDI: Indigenous participant Identifier
NIDI: Non-Indigenous participant Identifier
PAH: Princess Alexandra Hospital
WF: Wendy Foley
