# Supplementary material for: “I’m outta here!”: a qualitative investigation into why Aboriginal and non-Aboriginal people self-discharge from hospital
Source: BMC Health Serv Res. 2021 Sep 3;21:907. doi: 10.1186/s12913-021-06880-9 (PMC8414851; doi:10.1186/s12913-021-06880-9)
Supplement: Supplementary file 1 — Additional file 1. DAMA Study: Indigenous Hospital Liaison Office Interview Schedule [file 12913_2021_6880_MOESM1_ESM.doc]

**DAMA Study: Indigenous Hospital Liaison Office Interview Schedule**

1. What is the role of a HLO?

*Prompts:*

Tell us about a normal day in your role

Tell us about some exceptional things that happen

1. Do you have any involvement in the DAMA process? If yes, what is your role?

*Prompts:*

Who calls you? Nurse/doctor/ward receptionist?

What do they hope you can do?

What do you do?

How often are you involved in DAMA situations?

1. Can you give me an example of a DAMA situation that sticks out in your minds?
2. How often do you think patients DAMA? Are you aware of any wards where DAMA is more frequent then others? If yes, why do think this happens?
3. Do you think Indigenous people DAMA more then non-Indigenous people?
4. In your opinion, what makes people DAMA? (medical jargon, staff attitudes, boredom, distrust)
5. From your experience, what do you think hospital staff think of patients who DAMA? What about if these patients then return for further treatment?
6. From your experience, how do hospital staffs treat patients who have discharged AMA, then returned for further treatment?
7. What do you think about DAMA? Do you think it matters if patients DAMA? Please explain your answer. What do you think can be done to stop patients discharging AMA?
