# Supplementary material for: “I’m outta here!”: a qualitative investigation into why Aboriginal and non-Aboriginal people self-discharge from hospital
Source: BMC Health Serv Res. 2021 Sep 3;21:907. doi: 10.1186/s12913-021-06880-9 (PMC8414851; doi:10.1186/s12913-021-06880-9)
Supplement: Supplementary file 2 — Additional file 2. DAMA study: participant interview schedule [file 12913_2021_6880_MOESM2_ESM.docx]

**DAMA study: participant interview schedule**

1. **can you please tell us about the time that you went to hospital and left before the doctors thought that you were ready to be discharged?**

Prompt questions

- 1. Why did you have to go to hospital?
  2. How long were you in hospital for?
  3. What was it like being in hospital?
  4. What were the staff like – were they friendly, kind, caring? Did you feel listened to?
  5. Indigenous participants only – Did you see the Indigenous Hospital Liaison Officer? If yes, did they provide you with the support you needed? If no, were you told there was an Indigenous Hospital Liaison Officer who could come and see you if you wanted? Would you have liked to speak with the HLO?
  6. Did you feel that you knew what was going on with your treatment/recovery/procedures?
  7. are you a smoker? if yes, did the staff offer you any nicotine replacement therapy?

1. **The records say that you left hospital before the doctor had discharged you.**
   1. Why did you decide to do this?
   2. Did you tell anyone you were leaving? If yes, did they try to make you stay? Did they provide you with any information or advice about how to look after yourself; about going back to your GP; or when you should come back to hospital?
2. **Was this the first time that you’d been in hospital?**
   1. If no, how many other times and for what sort of reasons?
   2. Did you leave before the doctor had discharged you on any of these occasions? Why/why not?
   3. If no, what was different about this latest time?
3. **What do you think would have been needed to change in order for you to have stayed in hospital until the doctors thought that you were ready to be discharged?**
4. **Do you think that it is a problem if people discharge themselves from hospital? Why/why not?**
